# Myeloablative Anti-CD20 Radioimmunotherapy +/- High-Dose Chemotherapy Followed by Autologous Stem Cell Support for Relapsed/Refractory B-Cell Lymphoma Results in Excellent Long-Term Survival

**DOI:** 10.18632/oncotarget.1037

**Published:** 2013-06-12

**Authors:** Julia Y Wagner, Kathleen Schwarz, Susanne Schreiber, Burkhard Schmidt, Hans-Jürgen Wester, Markus Schwaiger, Christian Peschel, Christoph von Schilling, Klemens Scheidhauer, Ulrich Keller

**Affiliations:** ^1^ III. Medical Department, Technische Universität München, Munich, Germany; ^2^ Hematology and Oncology, Klinikum Freising, Freising, Germany; ^3^ Nuclear Medicine Department, Technische Universität München, Munich, Germany; ^4^ Pharmaceutical Radiochemistry, Technische Universität München, Garching, Germany

**Keywords:** Non-Hodgkin lymphoma, Radioimmunotherapy, CD20, High-dose chemotherapy, Autologous stem cell transplantation

## Abstract

**Background:**

Radioimmunotherapy (RIT) has been used to treat relapsed/refractory CD20+ Non-Hodgkin lymphoma (NHL). Myeloablative anti-CD20 RIT followed by autologous stem cell infusion (ASCT) enables high radiation doses to lymphoma sites. We performed a phase I/II trial to assess feasibility and survival.

**Methods:**

Twenty-three patients with relapsed/refractory NHL without complete remission (CR) to salvage chemotherapy were enrolled to evaluate RIT with Iodine-131 labelled rituximab (^131^I-rituximab) in a myeloablative setting. Biodistribution and dosimetric studies were performed to determine ^131^I activity required to induce a total body dose of 21-27Gy to critical organs. In 6/23 patients RIT was combined with high-dose chemotherapy. 8/23 patients received a sequential high-dose chemotherapy with a second ASCT. The median follow-up is 9.5 years.

**Results:**

6.956-19.425GBq of ^131^I was delivered to achieve the limiting organ dose to lungs or kidneys. No grade III/IV non-hematologic toxicity was seen with RIT alone. Significant grade III/IV toxicity (mucositis, fever, infection, one therapy related death) was observed in patients treated with RIT combined with high-dose chemotherapy. The overall response rate was 87% (64% CR). The median progression-free (PFS) and overall survival (OS) is 47.5 and 101.5 months. An international prognostic index score >1 was predictive for OS.

**Conclusion:**

Myeloablative RIT with ^131^I-rituximab followed by ASCT is feasible, well-tolerated and effective in high risk CD20+ NHL. Combination of RIT and high-dose chemotherapy increased toxicity significantly. Long-term results for PFS and OS are encouraging.

## INTRODUCTION

Non-Hodgkin lymphomas (NHL) comprise a heterogeneous group of B or T cell malignancies with a wide range of aggressiveness [1]. Current first-line options for advanced-stage indolent B-cell lymphoma include the unconjugated anti-CD20 monoclonal antibody rituximab either as single-agent or in combination with chemotherapy. Despite initial response to standard therapy a high proportion of patients with indolent NHL will ultimately develop disease progression. Treating relapsed or refractory indolent NHL is challenging, as there is no standard therapy defined. High-dose chemotherapy with autologous stem cell transplantation (ASCT) provides a treatment option enabling improved progression-free survival (PFS) although usually considered not to be curative in patients with indolent or transformed NHL including mantle cell lymphoma (MCL) [2,3]. Patients with minor or partial response to salvage chemotherapy prior to myeloablative treatment are at higher risk of relapse after short PFS compared to patients who are in complete remission (CR) or have minimal disease at the time of transplantation [2]. Additional therapy options for this high-risk patient population are therefore needed.

Radioimmunotherapy (RIT) uses monoclonal antibodies (mAb) directed against specific tumor antigens labeled with radioisotopes to deliver radiation directly to the tumor, thus combining synergistic effects of both radiation and immunotherapy with manageable local and systemic side effects [2]. Over 20 years ago successful use of an ^131^I-labeled anti B-cell lymphoma (Lym-1) mAb was reported for the first time in a patient with Richter's syndrome [2]. A few years later promising antitumoral efficacy of ^90^Y- and ^131^I-conjugated anti-CD20 mAb in B-NHL was described [3, 4]. Thereupon several studies using radionuclide-labeled anti-CD20 antibodies in non-myeloablative doses showed high response rates in patients with recurrent or refractory indolent lymphomas [5-10]. Only a minority of these remissions is however durable [11], the majority of responding patients finally develop disease progression. To further improve efficacy of RIT and to provide long-time remissions, several strategies were tested. Myeloablative doses of RIT followed by either autologous or allogeneic SCT emerged as promising approach, based on the observation that recurrence rates after external beam radiation therapy are a function of the delivered radiation dose [12]. Several studies have shown promising PFS data with myeloablative doses of RIT in patients with recurrent B-NHL [13-15]. Myeloablative RIT compared favorably with high-dose chemotherapy concerning PFS and OS in patients with relapsed follicular lymphoma (FL) [16]. Combination of myeloablative RIT with high-dose chemotherapy resulted in promising remission rates in patients with relapsed or refractory MCL [17]. Apart from this, ^131^I-tositumomab as first-line treatment showed prolonged clinical and molecular remissions in patients with advanced FL [18]. Long-term follow-up of these patients showed a median duration of response of six years, with approximately 40% of patients remaining progression-free at ten years [19]. More recently consolidation of first-line remission with ^90^Y-ibritumomab tiuxetan in patients with advanced-stage FL proved highly effective in a randomized phase III trial not only leading to significantly prolonged PFS but also converting PR after induction treatment into CR in a substantial proportion of patients [20]. Similarly impressive results have been shown for consolidation with ^131^I-tositumumab after induction chemotherapy with CHOP in patients with previously untreated, advanced-stage FL [21]. ^131^I-tositumomab (Bexxar^®^) is only approved in the United States, while ^90^Y-ibritumomab tiuxetan (Zevalin^®^) is also available in Europe.

Here we present data from a phase I/II study evaluating a tandem therapy approach comprising myeloablative RIT with a ^131^I-conjugated anti-CD20 mAb (^131^I-rituximab) followed by high-dose chemotherapy with autologous stem cell support in heavily pretreated patients with relapsed or refractory B-NHL. We report on feasibility, clinical efficacy and risk factors associated with inferior outcome. This trial provides the longest follow-up for myeloablative RIT in patients with high-risk NHL to date showing highly encouraging long-term PFS and OS.

## RESULTS

### Patient and Disease Characteristics

Patient and lymphoma characteristics are shown in Table 1. The median age of the enrolled patients was 58 years (range: 31-67 years). Patients had received extensive pretreatment with a median of 3 prior regimens (range: 1-11 regimens). Sixty-five percent (15/23) of patients had indolent lymphoma (n=14: FL; n=1 marginal zone lymphoma [MZL]). Thirteen percent of patients (3/23) had aggressive lymphoma transformed from FL. Twenty-two percent of patients (5/23) had MCL. Ninety-one percent (21/23) of patients had stage III/IV disease. Twenty-two percent (5/23) had an elevated serum lactate dehydrogenase before RIT, and 22% (5/23) had bone marrow involvement. All patients had a performance status 0 or 1 (Eastern cooperative group score). The FLIPI scores for the FL patients were as follows: 0 (n=1), 1 (n=4), 2 (n=7) and 3 (n=2), and n=3 not available. The IPI scores calculated for all patients were: ≤1 in 48% of patients (11/23) and >1 in 52% of patients (12/23).

**Table 1:** Patient demographics and clinical characteristics

	No. of patients	%	Median (range)
	23	100	
Age			58 (31-67)
Sex Female Male	13 10	57 43	
Histological subtype FL MZL MCL	17 1 5	74 4 22	
Stage of disease at first diagnosis II III/IV	2 21	9 91	
Bulky disease	9	39	
Bone marrow involvement	5	22	
LDH normal elevated not available	15 5 3	65 22 13	
Prior Rituximab	12	52	
No. of previous regimens			3 (1-11)
Duration of response after last standard therapy (months)			7 (1-57)
Pre-RIT status PR SD PD	11 5 7	48 22 30	
FLIPI 0-1 2 >2	5 7 2	28 39 11	
IPI >1 ≤ 1	12 11	52 48	

FL, follicular lymphoma; MZL, marginal zone lymphoma; MCL, mantle cell lymphoma; LDH, lactate dehydrogenase; PR, partial response; CR, complete response; SD stable disease; PD, progressive disease; FLIPI, follicular lymphoma international prognostic index; IPI, international prognostic index.

### Dosimetry

The post-therapeutic whole-body half-life was 118 hours (median) with a range from 92 to 160 hours. As estimated by dosimetric studies the kidney was the dose-limiting normal organ. The MTD reached was 27 Gy for the dose-limiting organ (kidney, respectively lungs). According to the planned dose escalation the first 16 patients were treated on a phase I trial in cohorts of 4 patients with radiation doses of 21, 23, 25 and 27Gy to the critical normal organ. Seven patients were then treated on the 27Gy level within the phase II study part. Activities of administered ^131^I ranged from 7.0 to 19.4GBq. The median estimated radiation doses for kidney and lung were 28Gy (range 7-37 Gy) and 17.8Gy, respectively. The measured whole-body radiation dose ranged from 1.9 to 9Gy (median 4.9Gy). The calculated bone marrow radiation dose ranged from 2.1 to 10.3Gy (median 5.1Gy). Lymphoma uptake

### Early Toxicity

All patients experienced expected grade IV hematotoxicity after RIT (Table 2). ASCT was performed when the residual body activity had fallen below 0.222GBq. Patients required a median of 21 days (range: 14-32 days) between RIT and ASCT. In median 2 (range: 0-4) erythrocyte and 3 (range: 1-9) platelet transfusions were required per patient. The median time to leukocyte recovery (>1000/μl) was 11 days and to platelet recovery (>20000/μl) 12 days after administration of RIT. Intravenous antibiotics were administered to 3 patients for treatment of neutropenic fever (≥38.3°C) without septic complications. No significant renal toxicity associated with RIT was observed. Non-hematological toxicity is listed in Table 2.

**Table 2:** Early toxicity

Toxicity	RIT n=9 (%)	Sequential High-dose chemotherapy n= 8 (%)	Combined RIT + High-dose chemotherapy n=6 (%)
Leukopenia/thrombocytopenia (grade III/IV)	9 (100)	8 (100)	6 (100)
Mucositis grade I/II/III/IV	1/0/0/0 (11)	0/4/2/1 (88)	0/1/2/1 (67)
Neutropenic fever	3 (33)	6 (75)	2 (33)
Pneumonia	0	2 (25)	0
Sepsis	0	0	4 (67)
TRM	0	0	1 (17)

RIT, Radioimmunotherapy; TRM, treatment related mortality.

Nine of 23 patients received a second myeloablative therapy using the BEAM protocol followed by a second ASCT. Four patients refused further myeloablative treatment and 5 patients were not eligible for a second myeloablative therapy due to disease progression after RIT. Two of them underwent allogeneic stem cell transplantation and the remaining 3 patients received percutaneous radiation or conventional chemotherapy. Toxicity of the myeloablative BEAM protocol was acceptable as expected in accordance with the literature [24] and is summarized in Table 2.

Due to reaching the primary endpoints of the phase I part of the study and compliance problems regarding the second high-dose chemotherapy, the remaining 5 of 23 patients were treated with a combined RIT plus high-dose chemotherapy approach, a strategy supported by encouraging data from the Seattle group [17]. In this group, 4 patients with MCL and one with transformed FL received high-dose chemotherapy (EAM protocol) with ASCT subsequent to RIT after the residual body activity had fallen below 0.222GBq. ASCT was performed one day after completion of chemotherapy in this schedule. Time from RIT to transplantation was slightly longer in this cohort (median 24 days, range 20-28 days). The time to hematopoietic reconstitution was comparable with the RIT only cohort. Two patients developed neutropenic fever and 4 patients presented with septicemia. Four patients showed mucositis grade II-IV. One of the patients treated with RIT in combination with high-dose chemotherapy EAM suffered a treatment related death (sepsis with multiple organ failure).

In conclusion, early non-hematologic toxicity occurred more frequently and was more severe in patients treated with RIT either in combination with EAM or with sequential BEAM followed by ASCT, as compared to myeloablative RIT alone. The higher grade III/IV toxicity was correlated with the combined RIT plus high-dose chemotherapy approach (p=0.005) and the incidence of mucositis (p=0.022).

### Response and Survival

Twenty-two of 23 patients were assessable for response. One patient was excluded due to therapy-related death after RIT plus EAM. The therapeutic outcome is summarized in Table 3. None of the patients was in CR prior to myeloablative RIT and all patients were extensively pretreated. The overall response rate (ORR) was 87% with 64% of patients (14/22) achieving a CR and 23% (5/22) achieving a PR. One patient had stable disease and 2 patients had progressive disease at the initial restaging upon completion of therapy. The pre-transplant status of the lymphoma did not influence response. As shown in Figure 2 the median OS of all patients was 101.5 months and the median PFS was 47.5 months, respectively, with a median follow-up of 9.5 years (range: 6.2-12.2 years). Median PFS after the last standard chemotherapy was 7 months. The OS and PFS separated for the RIT, RIT/EAM, and RIT/BEAM groups as well as for the RIT only group versus the groups having received RIT combined with EAM or RIT and BEAM sequentially is illustrated in Figure 3. Due to small sample size the differences in OS and PFS as shown in Figure 3 were not statistically significant. At the end of follow-up, 2 patients with complete or partial remission (1 PR, 1 CR with relapse after 2 years) had proceeded to allogeneic SCT. One patient died from multiple organ failure after allogeneic SCT and one is alive. One of the 23 patients was lost to follow-up. Forty-one percent of patients (9/22) are still in remission and 45% (10/22) are alive. All CR Patients were negative as assessed by FDG-PET 6 weeks after treatment. Patients in CR after myeloablative RIT had a significantly longer PFS and OS than non-CR patients (p=0.0009 and p=0.017).

**Table 3:** Response to treatment

Type of myeloablative treatment (n)	Disease status prior RIT n (%)	Response after RIT n (%)	Conversion Rate %
All (n=23*) CR PR SD PD	0 11 (48) 5 (22) 7 (30)	14 (64) 5 (23) 1 (4) 2 (9)	70 100 86
RIT alone (n=9) CR PR SD PD	0 4 (44) 2 (22) 3 (34)	3 (33) 4 (45) 0 2 (22)	78
RIT/BEAM sequential (n=8) CR PR SD PD	0 4 (50) 3 (37) 1 (13)	6 (75) 1 (12,5) 1 (12,5) 0	89
Combined RIT/High-dose chemotherapy (n=6) CR PR SD PD	0 3 (50) 0 3 (50)	5 (100) 0 0 0	100

* One patient was not analyzed for outcome after RIT and rate of conversion due to early death. RIT, Radioimmunotherapy.

### Risk Factors and Late Effects

The influence of different disease and treatment variables on PFS and OS was examined using univariate analyses. Results are summarized in Table 4. An elevated LDH immediately before initiation of RIT was a significant negative predictor for both PFS and OS (p=0.002 and p=0.003). An IPI>1 was a significant negative predictor for OS from the time of RIT respectively (p=0.018). Other parameters tested had no significant impact on OS/PFS. ECOG and extranodal involvement were not analyzed since the ECOG was ≤1 in all patients and no patient had involvement of >1 extranodal sites. One patient developed acute myeloid leukemia (AML) 5 years after myeloablative RIT without additional high-dose chemotherapy and died from AML with the lymphoma still in CR. Two patients developed therapy-related myelodysplasia (tMDS). One was diagnosed 10 years after myeloablative RIT alone and died 4 months later. The second patient developed tMDS 9.5 years after RIT/BEAM and underwent allogeneic SCT thereupon. At the end of follow-up he was alive and well.

**Table 4:** Univariate analysis for factors influencing OS and PFS

Variable	P		OS	PFS	Hazard ratio		OS	PFS	95 % CI		OS	PFS
Preinfusion of cold rituximab	0.766	0.739	0.841	0.841	0.269-2.623	0.304-2.327
No. of prior regimens	0.094	0.249	1.209	1.133	0.968-1.510	0.916-1.401
Duration of last response prior relapse/progression	0.299	0.183	0.946	0,938	0.852-1.050	0.853-1.031
PET status prior RIT	0.985	0.828	1.012	1.133	0.285-3.592	0.369-3.476
Histology	0.66	0.914	0.801	0.954	0.298-2.152	0.406-2.24
Hemoglobin prior RIT	0.71	0.589	0.748	1.372	0.161-3.467	0.435-4.323
LDH prior RIT	0.003	0.002	12.701	10.149	2.367-68.15	2.278-45.21
FLIPI	0.078	0.095	1.975	1.807	0.928-4.206	0.902-3.623
IPI (≤1 vs. > 1)	0.018	0.065	6.395	2.767	1.367-29.91	0.938-8.158
BM involvement	0.755	0.147	1.398	3.287	0.171-11.44	0.657-16.44
LN sites involved	0.261	0.390	0.307	0.52	0.039-2.410	0.117-2.313

OS, overall survival; PFS, progression-free survival; CI, confidence interval; PET, positron emission tomography; RIT, radioimmunotherapy; LDH, lactate dehydrogenase; FLIPI, follicular lymphoma international prognostic index; IPI: international prognostic index; BM, bone marrow; LN, lymph node.

## DISCUSSION

Although combination of rituximab with established chemotherapy regimens has improved ORR, PFS and OS in patients with newly diagnosed indolent or MCL [25] as well as in patients with diffuse large B-cell lymphoma [26, 27], prognosis of relapsed or refractory B-NHL is often poor. RIT has proved beneficial not only for patients with recurrent or refractory indolent or transformed B-NHL [28], but also as first-line treatment [18] and consolidation of first remission in previously untreated FL [20]. Subsequently ^131^I-tositumumab and ^90^Y-ibritumomab tiuxetan have been approved for treatment of relapsed or refractory indolent or transformed B-NHL. ^90^Y-ibritumomab tiuxetan furthermore received approval as consolidation therapy after remission induction in previously untreated FL. Despite these encouraging results RIT is infrequently used [29].

Primary aim of this trial was to evaluate the patient specific activity of ^131^I-rituximab in a myeloablative setting as salvage therapy prior to conventional high-dose chemotherapy with ASCT. Early trials have established an MTD of 27Gy to the critical organ lung when RIT was followed by ASCT [14]. In combination with high-dose chemotherapy followed by ASCT the Seattle group showed that 25Gy could be safely administered [30]. The delivered therapeutic dose is dependent on uptake and residence time of the radiolabelled antibody in normal organs like liver, lung and kidney as measured in dosimetric studies. In comparison with ^131^I-tositumomab the critical normal organ in our study was not the lung but mainly the kidney [31]. The median dose administered to the lung was just 17.8Gy, and accordingly we did not observe pulmonary toxicity. Despite some differences in the study design, the measured whole body doses were comparable in both studies, being 4.9Gy (range 1.9-9Gy) with ^131^I-rituximab versus 4.1Gy (range 2.6-10Gy) with ^131^I-tositumomab [13]. Based on earlier studies lymphoma uptake should be likely high [32].

Our study with 23 patients demonstrated that myeloablative RIT with or without chemotherapy is feasible and effective even in a heavily pretreated patient population with relapsed or refractory CD20^+^ B-NHL. A specific feature of this trial is the comparison of patients treated with RIT in combination with high-dose chemotherapy with patients receiving a tandem concept including RIT and high-dose chemotherapy followed by a second ASCT. Beyond that our trial provides the longest follow-up for RIT in high-risk NHL to date. Median PFS had been 7 months after the last chemotherapy that preceded RIT. After myeloablative RIT the median PFS was 47.5 months, clearly comparing favorable with data from previous studies[31, 33, 34]. The long-term outcome with regard to OS is encouraging with 43% of patients (10/23) being alive and 39% (9/23) still in CR after a median follow-up period of 9.5 years. These results are impressive considering the fact that all patients in this trial had experienced failure or relapse of their disease prior to study entry and were heavily pretreated. It however should be considered that about 48% of the participating patients were Rituximab naive, which most likely had an impact on PFS and OS rates achieved after RIT.

The majority of patients in this trial had FL. Currently there is no defined standard approach for relapsed/refractory FL. For eligible patients myeloablative chemotherapy (e.g. the BEAM protocol) followed by ASCT is an established treatment. Supporting data come from several trials in FL and suggest to especially considering patients with poor initial response, response duration less than the mean PFS for the respective treatment regimen, and/or a high FLIPI score [35-38]. Total body irradiation plus cyclophosphamid (TBI-Cy) was also used as a preparative regimen followed by ASCT. However, a possible association between TBI-Cy and a significantly higher incidence of fatal treatment-related MDS/AML was observed [38], whereas RIT did not exceed average rates of secondary malignancies [39]. These data and inherent high radiosensitivity of FL combined with expression of target antigens make RIT a promising concept in the treatment of FL. As myeloablative RIT is safe and feasible when followed by ASCT with low incidence of secondary malignancy it is a reasonable alternative regimen especially in elderly patients and patients who have concerns about high-dose chemotherapy.

The IPI score determined at the time of diagnosis evolved as relevant factor for outcome in our patient group. Prognosis of patients with more than one risk factor (IPI>1) was significantly worse compared to patients with IPI≤1. Our finding that myeloablative RIT could not eliminate these differences are supported by the data of Solal-Celigny and colleagues[40].

A possible advantage for the tandem concept tested in this study remains unclear due to the limited total patient number. Eight patients were treated with the tandem therapy approach, 9 patients with myeloablative RIT only. In comparison to the RIT only patients the CR rate was higher in the tandem therapy population (33% versus 75%). However, all CRs in the tandem therapy group were achieved already after myeloablative RIT, not after subsequent high-dose chemotherapy, and the RIT only patients in our study had a rather low CR rate (33%) compared to earlier trials that applied myeloablative RIT and achieved CR rates of approximately 50% [41]. Although there are no statistically significant differences between RIT and RIT in combination with high-dose chemotherapy with or without a second ASCT, there is a trend suggesting that RIT in combination with high-dose chemotherapy could be a reasonable approach for improving long-term PFS and OS.

Early non-hematologic toxicity after RIT in combination with high-dose therapy was significantly higher as compared to RIT only, including one treatment-related death. One other major concern with regard to high-dose chemotherapy/ASCT is the possibility of inducing treatment-related secondary malignancies. Three patients (13%) in our study developed secondary MDS or AML, a proportion that is comparable with other studies[39, 42].

At present RIT is underutilized in routine practice despite approval by the responsible regulatory authorities in the United States and in Europe. Treating physicians seem hesitant to recommend RIT to a larger number of potentially eligible patients. This might be due to availability of alternative non-radioactive therapies and various logistic, educational and economic concerns[43]. However, besides this study, recent data from Leahy and Turner demonstrated that RIT with ^131^I-rituximab in routine clinical outpatient practice provides a safe and cost-effective treatment option for relapsed or refractory indolent NHL with half of patients achieving a durable CR with potential for repeat[44]. Recently a possible role of RIT as part of the conditioning regime prior to allogeneic SCT in patients with persistent high-risk B-NHL was evaluated. In two phase II trials RIT proved feasible and safe in combination with a reduced-intensity conditioning regime consisting of fludarabine and TBI (2Gy) with acceptable toxicity even in elderly and heavily pretreated patients [45, 46]. These results and our data in the autologous setting provide an important perspective for future RIT studies.

## MATERIALS AND METHODS

### Patients

All 23 Patients had histologically confirmed relapsed or refractory CD20+ NHL and were enrolled in this prospective single-center phase I/II study between January 2000 and October 2004. The study protocol was approved by the ethics committee of the medical faculty of the Technische Universität München. All participating patients signed a written informed consent. Patient demographics and clinical characteristics are shown in Table 1. Inclusion criteria were: histologically confirmed diagnosis of CD20-expressing, advanced B-NHL; pretreatment with at least one standard chemotherapy protocol and disease progression; lack of response with measurable residual disease or a recurrence with inadequate response to the reinduction therapy; a collection of 2×10^6^ CD34+ cells/kg body weight (BW) per planned transplantation; confirmed uptake of ^131^I-rituximab by lymphoma tissue; age ≥18 and ≤65 years; life expectancy of at least 3 months; performance status of 60 or higher on the Karnofsky scale; leukocytes > 3500/μl, granulocytes > 1500/μl, platelets >10^5^/μl, hemoglobin (Hb) >10g/dl. Minimal requirements for organ function included myocardial function with Fractional Shortening >33% on echocardiography; pulmonary function with diffusion capacity for carbon monoxide >50%; negative pregnancy test and adequate contraceptive measures during and one year after completion of the study.

### Treatment Plan

The primary study endpoints were to identify the appropriate patient-specific activity of ^131^Iodine-labeled rituximab to be administered at maximum tolerated dose (MTD) level in a myeloablative setting as salvage therapy prior to high-dose chemotherapy with ASCT and to evaluate biodistribution of the conjugated antibody by pretreatment with increasing doses of the unlabeled antibody. The MTD of ^131^I-rituximab was based on absorbed radiation doses to critical organs (lung and kidney). Secondary study objectives were determination of response to treatment, PFS and OS. The median time from initial diagnosis to beginning of RIT was 28 months (range: 5 to 235 months).

**Figure 1:**
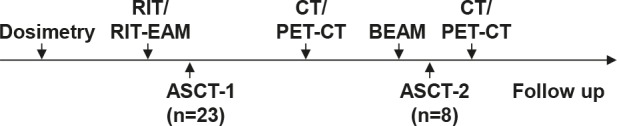
Treatment schedule

Since the lung is the dose-limiting organ (27.25Gy organ dose) besides the bone marrow (*12*), this study tested lung exposure in cohorts consisting of 4 patients with increasing doses of 21, 23 and 25Gy. The MTD was reached if 2 of 4 patients of a cohort showed grade IV non-hematological toxicity. If grade III toxicity was observed in 3 patients from a cohort, a second cohort of 2 patients was enrolled at the identical dose level. If one additional grade III toxicity occurred in this cohort, MTD was reached. If no further dose limiting toxicity or severe adverse event was observed, further dose-escalation to the next cohort was permitted. A summary of the whole treatment schedule is depicted in Fig. 1.

**Figure 2:**
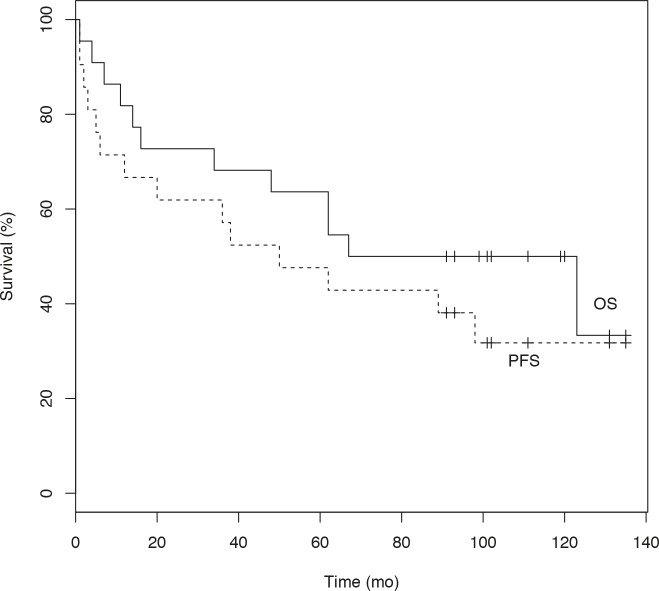
Overall survival (OS) and Progression-free survival (PFS) in all participating patients

**Figure 3:**
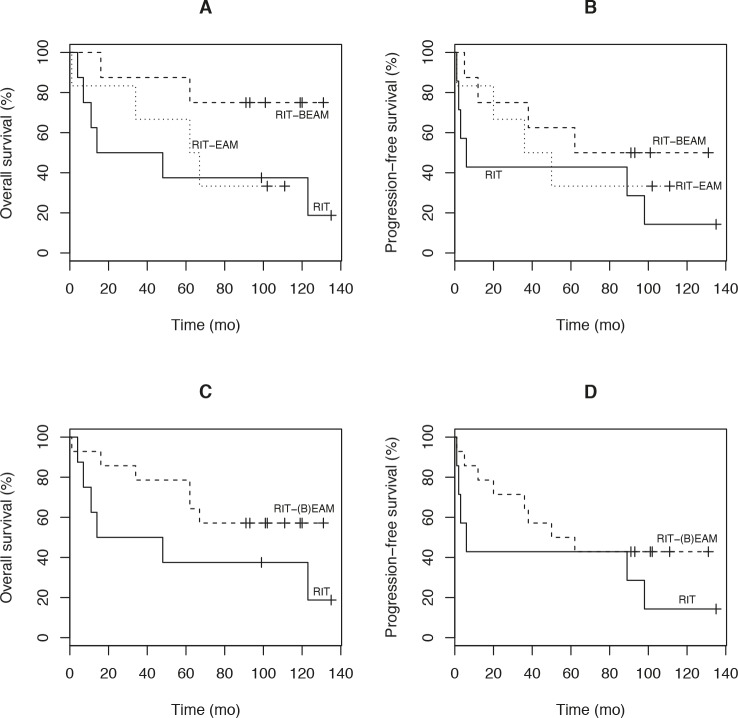
(A) Overall survival and (B) Progression-free survival according to treatment modality. (C) Overall survival and (D) Progression-free survival of patients who received RIT alone versus patients who received combined RIT/HD-CTX or RIT/BEAM sequentially

### Radiolabelling and Dosimetry

^131^I radiolabelling of rituximab was performed as described earlier [22]. Briefly, Rituximab (10mg/ml) was labeled with iodination grade 131I-Iodide in an Iodogen coated PP vial (20 ml) in PBS buffer. After labeling, the entire solution was purified by size exclusion chromatography and formulated in isotonic saline by sterile filtration. Quality control was carried out by thin layer chromatography resulting in >98% protein bound radioactivity. After completion of mobilization therapy and baseline investigations, a biodistribution study was conducted on each qualifying patient before treatment in order to determine the total activity to be applied. Each patient received at least one time a dose of 2.5 mg/kg BW cold rituximab before therapy. For thyroid blockade each patient received cold iodine (Lugol's solution) starting 1 to 2 days before activity application and continued until 3 weeks thereafter. The ^131^I-labeled anti-CD20 antibody rituximab (185-370 MBq) was infused over a period of 30-60 minutes in a volume of 100 ml of 0.9% NaCl containing 5% human serum albumin. Immediately after infusion of the calibrated activity of ^131^I-rituximab each patient was measured in anterior and posterior positions. Simultaneous anterior and posterior whole-body scans (conjugate views) were obtained immediately after the first probe measurement using a large field-of-view dual-head γ-camera (Vertex, ADAC Laboratories, USA). γ-camera measurements were repeated 24, 48, 72, 120 and up to 168 hours after infusion. Regions of interest (ROI) were defined for whole-body, heart, liver, lung, kidney, spleen, and background. Organ activity curves were fitted to a monoexponential function. From this fit the effective half-life and the uptake 0.5 hours after infusion was derived. The activity uptake (A_0_) 0.5 hours after infusion. was normalized to the injected activity A_inj_ and the residence time calculated according to τ = A_0_/A_inj_*T_1/2 eff_/ ln 2, which were used as input to the MIRDOSE 3 program.

### Myeloablative Radioimmunotherapy +/− High-Dose Chemotherapy EAM

Patients received a therapeutic infusion of 7.0 to 19.4GBq radiolabeled antibody (40mg rituximab). Activities to be used were calculated from the dosimetric studies according to the predetermined dose for the normal critical organ assigned to the patient's cohort. The effective therapeutic total body dose (TBD) was calculated from whole-body probe measurements. According to German radiation safety guidelines, precautions were taken as are required when applying high-dose radioiodine therapy in thyroid cancer patients. After sufficient activity decrease a whole body scintigraphy was performed to reconfirm the therapeutic biodistribution of the radiolabeled antibody compared to the dosimetric studies.

Subsequently patients received ASCT in the hematology department. Until hematopoietic reconstitution they remained hospitalized. Reconstitution was supported by daily subcutaneous applications of G-CSF (Neupogen^®^, 5μg/kg BW) starting at day 5 after ASCT. In 6 of 23 patients RIT was combined with high-dose chemotherapy EAM (etoposide 200mg/m^2^, twice daily from d-7 to d-3, cytarabine 500mg/m^2^, twice daily from d-7 to d-3 and melphalan 140mg/m^2^, d-1, all given intravenously). ASCT was performed on day 0.

### Myeloablative RIT with Subsequent Conventional High-Dose Chemotherapy Followed by Second ASCT

In 8 of the remaining 17 patients who had been treated with myeloablative RIT without EAM, a second myeloablative therapy was conducted at an 8-12 weeks interval after RIT. It was up to the investigator to administer this second myeloablative therapy or not. These 8 patients received high-dose chemotherapy using the BEAM protocol with BCNU (300mg/m^2^ d-7), etoposide (200mg/m^2^, twice daily from d-7 to d-3), cytarabine (500mg/m^2^, twice daily from d-7 to d-3) and melphalan (140mg/m^2^, d-1), all given intravenously. The second ASCT was performed on day 0 and patients remained hospitalized until hematopoietic reconstitution, again supported by daily subcutaneous applications of G-CSF (Neupogen^®^, 5μg/kg BW) starting at day 5 after ASCT.

### Response Criteria, Staging and Follow-up

Responses were graded according to standardized criteria as follows. Complete remission: complete regression of all measurable tumor parameters and clinical tumor signs; Partial remission: a reduction of at least 50% in the sum of the diameters of measurable disease; no appearance of new tumor lesions; no increase in size of any tumor lesion; Stable disease: reduction of less than 50% in the sum of the diameters of lesions without increase in size of any lesion and without new lesions; Progressive disease: increase in size greater than 25% of any lesion, appearance of new tumor lesions or both of these criteria. Toxicity and adverse events were graded using the National Cancer Institute Common Terminology Criteria of Adverse Events version 3.0. Median follow-up was 114 months. Disease staging using computed tomography (CT) and positron emission tomography (PET) scans was performed at the time of study entry. CT was repeated during follow-up every three months (first year), every six months (year 2-3) and yearly thereafter. FDG-PET study in addition to the RECIST relevant method (CT) was done at six weeks after treatment in all patients. Extensive laboratory tests including human anti-mouse antibody (HAMA) and a bone marrow biopsy were performed at baseline and repeated at regular intervals during the study period and during follow-up.

### Statistical Analysis

All time periods were calculated in months. The Kaplan-Meier method was used to calculate OS and PFS. Response definition followed the recommendations of the International Workshop NHL Response Criteria [23]. OS was calculated from initiation of RIT until death from any cause. PFS was calculated from administration of RIT until relapse or disease progression. The log-rank test was used to compare survival curves. The univariate Cox-PH-regression model was used to estimate hazard ratios and their 95% CI.

## CONCLUSION

Our study clearly suggests that myeloablative RIT provides long-term remission and survival in a substantial proportion of patients with relapsed or refractory *B-NHL*. The encouraging data of this long-term follow-up is opposing to the infrequent use of RIT followed by ASCT in the described patient population. Further investigation with an increased number of patients is needed to evaluate the significance of the combination approach with myeloablative RIT alone or in combination with high-dose chemotherapy and a second ASCT.
